# Prevalence of Liver Steatosis in Tuberous Sclerosis Complex Patients: A Retrospective Cross-Sectional Study

**DOI:** 10.3390/jcm13102888

**Published:** 2024-05-14

**Authors:** Thaïs De Bock, Carola Brussaard, Silke François, Karlien François, Laura Seynaeve, Anna Jansen, Karl Martin Wissing, Peter Janssens

**Affiliations:** 1 Department of Nephrology and Arterial Hypertension, Universitair Ziekenhuis Brussel (UZ Brussel), Vrije Universiteit Brussel (VUB), 1090 Brussels, Belgium; thais.de.bock@vub.be (T.D.B.); karlien.francois@uzbrussel.be (K.F.); karlmartin.wissing@uzbrussel.be (K.M.W.); 2 Department of Radiology, Universitair Ziekenhuis Brussel (UZ Brussel), Vrije Universiteit Brussel (VUB), 1090 Brussels, Belgium; carola.brussaard@uzbrussel.be; 3 Department of Gastroenterology, Universitair Ziekenhuis Brussel (UZ Brussel), Vrije Universiteit Brussel (VUB), 1090 Brussels, Belgium; silke.francois@uzbrussel.be; 4 Department of Neurology, Universitair Ziekenhuis Brussel (UZ Brussel), Vrije Universiteit Brussel (VUB), 1090 Brussels, Belgium; laura.seynaeve@uzbrussel.be; 5 Department of Pediatric Neurology, Universitair Ziekenhuis Antwerpen (UZA), 2650 Antwerpen, Belgium; anna.jansen@uza.be

**Keywords:** TSC, mTOR, liver steatosis, liver angiomyolipomata, MRI

## Abstract

**Introduction:** Tuberous sclerosis complex (TSC) is a genetic disease caused by pathogenetic variants in either the *TSC1* or *TSC2* genes. Consequently, the mechanistic target of the rapamycin complex 1 (mTORC1) pathway, a regulator of cell growth, metabolism, and survival, becomes inappropriately activated, leading to the development of benign tumors in multiple organs. The role of mTORC1 in lipid metabolism and liver steatosis in TSC patients has not been well-studied, and clinical data on liver involvement in this population are scarce. **Methods:** We conducted a retrospective, cross-sectional study to compare liver steatosis in TSC patients with age-, sex-, BMI-, and diabetes status-matched controls. Participants with a definite diagnosis of TSC were recruited from the TSC clinic at UZ Brussel. Liver steatosis was quantified using the fat signal fraction from in-phase and out-of-phase MRI, with a threshold of ≥5% defining the presence of steatosis. We also evaluated the prevalence of liver angiomyolipomata in the TSC group and analyzed risk factors for both liver steatosis and angiomyolipomata. **Results:** The study included 59 TSC patients and 59 matched controls. The mean fat signal fraction was 4.0% in the TSC group and 3.9% in the controls, showing no significant difference (two-tailed Wilcoxon signed ranks test, *p* = 0.950). Liver steatosis was observed in 15.3% of TSC patients compared to 23.7% of the controls, which was not statistically significant (two-tailed McNemar test, *p* = 0.267). Liver angiomyolipomata were identified in 13.6% of the TSC cohort. **Conclusions:** Our study, describing in detail the liver phenotype of TSC patients, did not reveal a significant difference in the prevalence of MRI-assessed liver steatosis in a large cohort of TSC patients compared to a closely matched control group.

## 1. Introduction

Tuberous sclerosis complex (TSC) is a rare, autosomal dominant disease characterized by proliferative lesions that impact multiple organs including the central nervous system, skin, heart, lungs, eyes, kidneys, and liver [1]. The disease arises from pathogenetic variants in either the *TSC1* or *TSC2* genes, leading to disruption of the TSC protein complex and subsequent hyperactivation of the mechanistic target of the rapamycin complex 1 (mTORC1) pathway, which drives increased protein synthesis and cellular proliferation [2]. mTOR-inhibitors (mTORi) have emerged as the cornerstone of TSC treatment, proving effective in the management of severe manifestations including subependymal giant cell astrocytomas (SEGAs) and kidney angiomyolipomata [3,4,5,6].

Liver steatosis, as observed in the context of metabolic dysfunction-associated steatotic liver disease (MASLD)—formerly known as non-alcoholic fatty liver disease (NAFLD)—presents a complex interplay of metabolic dysregulation. mTORC1, a central node in cellular metabolism that orchestrates anabolic processes including lipid synthesis, is under investigation for its intricate role in this context, with conflicting results [7,8]. Research has shown that liver-specific activation of mTORC1, via *TSC1* knockout in mice, confers protection against steatosis by modulating lipid export and synthesis [9,10]. In contrast, the inhibition of mTORC1 has been implicated in halting the progression of liver steatosis and inflammation in both murine models and human studies [11,12]. Clinically, the metabolic effects of mTORi are well-known and include elevated levels of cholesterol and triglycerides, insulin resistance and glucose intolerance, hypophosphatemia, and hyperuricemia [13]. In solid organ transplant patients, mTORi has been identified as a risk factor for the development of liver steatosis [14,15].

TSC presents a unique and valuable model for investigating the clinical ramifications of mTORC1 pathway dysregulation, offering insights that extend beyond the disease itself to broader biological and therapeutic implications. The liver phenotype of TSC remains underexplored: data on liver steatosis in animal models for TSC are scarce and studies on liver steatosis in humans with TSC are lacking.

The main objective of this study was to investigate the prevalence of liver steatosis, assessed with MRI, in patients with TSC in comparison to a control group matched for age, sex, BMI, and diabetes status. We assessed hepatic steatosis by measuring the MRI fat signal fraction, with a threshold of ≥5% to define steatosis. Potential risk factors for liver steatosis were explored. Additionally, the prevalence, characteristics, and risk factors associated with liver angiomyolipomata in TSC patients were assessed.

Altogether, the results of this research contribute to a more comprehensive understanding of the liver phenotype in TSC.

## 2. Methods

### 2.1. Study Design and Population

We performed a retrospective, matched-cohort, cross-sectional monocentric study. All procedures were approved by the ethical committee of UZ Brussel (EC-2022-252). Patient consent was waived due to the retrospective study design, where opting out applied. Inclusion and exclusion criteria are summarized in Table 1. Subjects with a definitive diagnosis of TSC (Supplementary Table S1), followed at the UZ Brussel outpatient TSC clinic, that received an in-phase and out-of-phase or DIXON abdominal MRI between March 2015 and June 2022 were included in this study. TSC patients followed at UZ Brussel without contraindications undergo routine abdominal MRI surveillance according to the guidelines [16]. Out of 102 TSC patients, 59 could be included. For each TSC patient, the most recent available image was used.

To minimize selection bias, controls without increased risk for liver disease were selected from the UZ Brussel abdominal MRI image repository through a prespecified structured selection protocol (detailed in the Supplementary Materials file). Briefly, potential controls were paired with TSC patients based on sex and within age brackets spanning three years. These pairs were then ordered by the closeness of their age at the time of imaging, prioritizing the closest matches. For an eligible match, the control’s body mass index (BMI) needed to be within ±5 kg/m^2^ of the TSC patient’s BMI, and their diabetes status had to be identical. In the final step, we applied exclusion criteria to the potential control subjects. When a control was deemed unsuitable, we proceeded to the next closest match in terms of age and sex until a suitable control was identified.

Clinical and demographic data from the electronic medical files were recorded using Research Electronic Data Capture (REDCap). An overview of the collected variables can be found in Supplementary Tables S2 and S3.

### 2.2. Image Acquisition and Endpoints

In-phase and out-of-phase MRI was used as a non-invasive method to determine the amount of liver steatosis. This technique is explained in Equation (1). To quantify liver fat, the fat signal fraction (η) was calculated. Liver steatosis is defined as a fat signal fraction of ≥5% on MRI [18].

(1) $$\eta =\frac{{SI}_{M(in)}-{SI}_{out}}{2\times {SI}_{M(in)}}$$

Formula to calculate the fat signal fraction. The fat signal fraction is calculated through in-out-in phase series with a 1.5 Tesla MRI. The signal intensity in phase is measured at 4.6 and 9.2 milliseconds. The mean of both is then calculated. The signal intensity out of phase is measured at 6.9 milliseconds. SI_M(in)_ stands for the mean signal intensity in phase and SI_out_ stands for the signal intensity out of phase. During the in-phase, the MRI is set to capture signals when the magnetic moments of both water and fat in the liver are synchronized, meaning that they are ‘in phase’. This produces a clear image that represents the combined signal of both water and fat, which can be useful for seeing the overall structure of the liver. Conversely, in an out-of-phase MRI, the imaging is timed to catch the moments when the magnetic properties of water and fat are out of sync, or ‘out of phase’. When this happens, the signals from fat cancel out some of the signals from water. This is especially helpful because areas with a lot of fat lose signal and appear darker on the MRI image. This contrast allows us to see how much fat is present in the liver [18].

**Figure 1:**
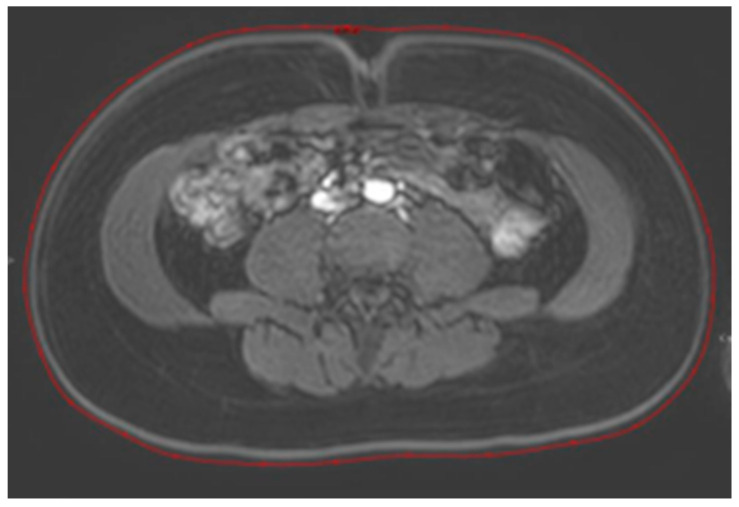
Measurement of the abdominal circumference on MRI. Measurements were made on axial images of the dynamic contrast series at the level of the umbilicus by surrounding the image of the skin (red line).

The abdominal circumference was also determined with an MRI. Measurements were made on axial images of the dynamic contrast series at the level of the umbilicus by surrounding the image on the skin, seen as a light grey line (see Figure 1).

Additionally, liver angiomyolipomata were evaluated. The number of lesions was recorded as well as the diameter of the largest angiomyolipoma. Liver angiomyolipomata were further graded using the system developed by Dabora et al. (Table 2) [19].

### 2.3. Statistical Analysis

All statistical analyses were conducted using IBM SPSS Statistics. Statistical analysis and two-tailed tests were used, and the test was considered statistically significant when the *p*-value was less than 0.05. The mean and standard deviation (SD) as well as the median and interquartile range (IQR) were calculated for continuous variables. Categorical variables were represented by frequency and percentage.

A dependent *t*-test was used to compare the normally distributed variables between the TSC subjects and matched controls. A Wilcoxon signed-rank test was used for the not normally distributed variables. Categorical data were evaluated using McNemar’s test.

The percentage of steatosis in TSC patients compared to the matched controls was analyzed via a Wilcoxon signed ranks test and the proportion of TSC patients compared to matched controls via the McNemar test. Conditional logistic regression analysis was used to analyze factors associated with liver steatosis as well as factors associated with liver angiomyolipoma in the TSC cohort. Considered pre-defined exposures entered as covariates in the conditional logistic regression model are listed in Supplementary Tables S2 and S3.

## 3. Results

In this study, 59 TSC patients and 59 matched controls were included. The characteristics of the TSC patients and controls are summarized in Table 3.

The frequency and percentage of the major diagnostic criteria present in patients with TSC are represented in Table 4, together with the results of the genetic analysis. The most frequent major diagnostic criteria in the studied TSC population were angiofibroma (66.1%), cortical tubers (94.9%), and subependymal nodules (66.1%). In 22%, a *TSC1* mutation was found, whereas 54.2% of TSC patients carried a *TSC2* mutation.

Mean abdominal circumference as measured on MRI was statistically higher in TSC patients compared to the controls (81.2 cm vs. 77.6 cm, *p* = 0.019). In addition, lower mean AST (26.7 U/L vs. 51.2 U/L, *p* = 0.003) and ALT (28.4 IU/L vs. 76.5 IU/L, *p* = 0.017) levels were found in the TSC group compared to the control group. The lipid profile between both groups was also compared. No significant difference could be found between the two groups. However, it is important to note that there was a considerable amount of missing data within the control group. TSC patients had frequent use of mTOR inhibitors (27.1%) and anti-epileptics (50.8%) whereas one control subject was on anti-epileptics.

**Figure 2:**
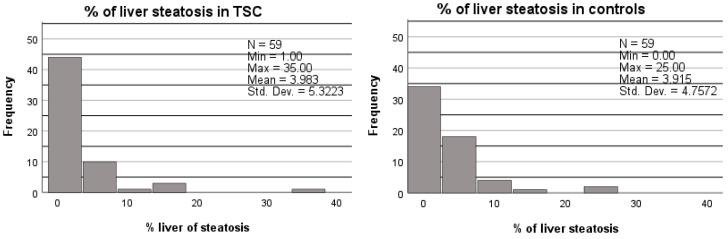
Percentage of liver steatosis in TCS patients compared to age, sex, BMI, and diabetes-matched controls. A Wilcoxon signed ranks test was used to compare the percentage of liver steatosis in both groups. No significant difference was found between the TSC patients and controls (*p* = 0.950).

No difference was found in the mean MRI fat signal fraction between the TSC subjects and matched controls (4.0% vs. 3.9%, *p* = 0.950; Figure 2 and Table 5). In addition, no difference was found in the proportion of liver steatosis between the TSC subjects and matched controls (9/59 vs. 14/59, *p* = 0.267; Table 5).

A conditional logistic regression analysis was used to examine potential risk factors associated with liver steatosis (Table 6). The univariate odds ratio (OR) of liver steatosis in patients with TSC was 0.44 (95% confidence interval: 0.14–1.44; *p* = 0.177) compared to the matched controls. Among patients with TSC, only one out of sixteen patients receiving mTORi therapy had liver steatosis whereas this was the case in eight out of forty-three patients without mTORi therapy (OR 0.29; 95% confidence interval: 0.033–2.54; *p* = 0.26). After adjustment for use of mTORi, the OR for liver steatosis associated with TSC increased to 0.75 (95% confidence interval: 0.17–3.35; *p* = 0.706). No difference in odds ratio was found after the adjustment of current medication intake. A total of 5/30 TSC patients on anti-epileptics had liver steatosis. The effect of anti-epileptic medication was not explored in the multivariate analysis, since only one person in the control group took anti-epileptic medication. The same applies to the effect of anti-hypertensive medication.

Upon discovering average elevations in the ALT and AST levels within the control group, further investigative efforts were undertaken to elucidate these unexpected findings. Twelve subjects, all in the control group, presented with elevated transaminase levels. As detailed in Supplementary Table S4, the increase could be attributed to the presence of gallstones in the majority of cases. A total of 3/12 of these subjects had liver steatosis.

A total of 13.6% of TSC patients had detectable liver angiomyolipomata on MRI. The median diameter of the largest liver angiomyolipoma was 0.7 cm and angiomyolipomata were of low grade (Table 7). Five TSC patients with liver angiomyolipomata had a pathological variation in *TSC2*, the other three were not genetically tested. Out of these eight patients with liver angiomyolipomata, four were female and four were male. The number of patients with angiomyolipomata was too small for a meaningful analysis of risk factors.

## 4. Discussion

We conducted a retrospective, cross-sectional study comparing TSC patients and age-, sex-, BMI-, and diabetes status-matched controls to evaluate the occurrence of liver steatosis in patients with TSC. No difference was found in the mean percentages of liver steatosis, nor in the proportion of liver steatosis between both groups. A total of 15.3% of the TSC patients and 23.7% of the matched controls had liver steatosis on abdominal MRI.

It remains unknown whether the systemic mTORC1 activation of TSC is protective, promotive, or neutral for the development of liver steatosis.

The gold standard for the diagnosis of steatosis is via liver biopsy and is defined as excessive liver lipid accumulation (triglyceride content of more than 5% of liver weight) [20]. However, MRI as a non-invasive method to determine the amount of steatosis is a well-accepted alternative. The prevalence of MASLD diagnosed on imaging (ultrasound, computed tomography scan, and magnetic resonance imaging/spectroscopy) is approximately 25% in the adult population [21,22,23]. This reported prevalence grossly corresponds to the percentage of hepatic steatosis found in our control population (23.7%), which was specifically selected to be a population with the absence of risk factors for liver steatosis, except for BMI and diabetes status according to matching needs. Stringent matching was performed to study whether TSC changes the risk for steatosis.

Although a trend towards a lower steatosis incidence in TSC patients was noted, we did not find a significant difference in the prevalence and percentage of liver steatosis in the studied TSC population compared to the control population. Several potential explanations for these findings can be suggested. First, methodological limitations need to be taken into consideration. This study was not sufficiently powered to detect subtle differences in steatosis prevalence. In addition, the selection of our control population does not guarantee that the sample is representative of the healthy general population, since all subjects had an MRI for a medical indication in this retrospective study (Supplementary Table S4). While the control group was stringently matched for risk factors for liver steatosis, transaminases were more elevated in this group. Symptomatic choledocholithiasis was the most frequent associated cause, a factor not directly considered a risk factor for the development of liver steatosis. Second, there was a high prevalence of mTORi use in the studied TSC population. It is interesting to note that only one out of sixteen (6%) TSC patients treated with mTORi therapy had liver steatosis, while this was the case in eight out of forty-three (19%) patients without mTORi. Although the small sample does not provide sufficient statistical power for hypothesis testing, this observation is compatible with a protective effect of mTORi against steatosis. Adjusting for mTORi therapy in conditional logistic regression analysis attenuated the trend to lower odds of liver steatosis in patients with TSC, suggesting that the observed trend might be to a large extent explained by the frequent use of mTORi in this population. It must be acknowledged that we cannot rule out that the observed results were simply the result of random error in the context of the relatively small sample size. Although only one patient in the control population took anti-epileptics, these molecules (e.g., valproic acid) can also increase the risk for steatosis [24]. Furthermore, the current study did not look at fibrosis, a factor important for long-term prognosis [25].

Little is known about liver angiomyolipomata in TSC patients. The exact prevalence is unclear, but liver angiomyolipomata have been described in up to 14.9% of TSC patients. Jozwiak et al. described an association between liver angiomyolipomata and *TSC2* mutations, co-existing renal angiomyolipomata as well as the fact that these lesions were more common in females compared to males [26]. In our studied TSC population, we did find similar results regarding the prevalence of liver angiomyolipomata (13.6%), and liver angiomyolipomata were indeed more frequent in patients with *TSC2* mutations. However, no association was found between liver angiomyolipomata and gender. Due to the small number of patients with liver angiomyolipomata (N = 8), the analysis was underpowered.

By grading liver angiomyolipomata according to Dabora et al., in our study population, we recognized that some patients could not be categorized. Therefore, we suggest a minor modification to this system by changing Grade 2 to ‘One or more liver angiomyolipomata, one or more > 1 cm diameter, and all < 4 cm in diameter’ instead of ‘Multiple liver angiomyolipomata, one or more > 1 cm diameter, and all < 4 cm in diameter’.

Most liver angiomyolipomata are described as nonprogressive and asymptomatic [26]. However, lesions may grow over time and symptomatic cases with abdominal pain, distention, and spontaneous bleeding of a liver angiomyolipoma have been described [27,28]. None of the studied patients presented liver angiomyolipoma-related bleeding. Few studies describe the effect of mTORi on the size of liver angiomyolipomata. One patient in our cohort was initially referred with a suspicion of liver metastasis, and lesions regressed on mTORi. Sirolimus has been described to be effective in reducing the size of liver angiomyolipomata in some published cases [29,30,31].

In conclusion, we did not find a difference in the incidence of liver steatosis assessed by MRI in a large TSC cohort compared to the matched controls. A possible protective effect of mTORi therapy on the development of liver steatosis should be assessed in an adequately powered study. Further investigations involving larger cohorts including a greater number of patients treated with mTOR inhibitors are warranted.

## Figures and Tables

**Table 1:** Inclusion and exclusion criteria for the selection of subjects. BMI: body mass index; HIV: human immunodeficiency virus; MRI: magnetic resonance imaging; TBC: tuberculosis; TSC: tuberous sclerosis complex. Alcohol abuse is defined as ≥30 g/day for men and ≥20 g/day for women; Uncontrolled hypertension is defined as an office blood pressure ≥ 140/90 mmHg [17].

	Inclusion Criteria	Exclusion Criteria
**TSC patients**	- Patients with a definite clinical or genetical diagnosis of TSC - Underwent in-phase and out-of-phase or DIXON abdominal MRI at UZ Brussels between April 2016 and June 2022	- Uncertain diagnosis of TSC - Data on BMI at ±1.5 years of imaging unavailable
**Controls**	- Underwent in-phase and out-of-phase or DIXON abdominal MRI at UZ Brussels between April 2016 and June 2022	- Data on BMI at ±1.5 years of imaging unavailable - MRI for indication of “suspicion of liver pathology” - Pregnancy - Chronic (>3 months) use of medication associated with the occurrence of steatosis, including amiodarone, diltiazem, tamoxifen, or steroids. - (A history of) chemotherapy - Alcohol abuse - Uncontrolled hypertension - History of viral hepatitis - Presence of hemochromatosis, TBC, auto-immune hepatitis, HIV - Familial dyslipidemia

**Table 2:** Grading system for angiomyolipomata in the liver developed by Dabora et al. [19].

*Grade*	*Number of Liver Angiomyolipomata*
*Grade 0*	None
*Grade 1*	One or more, all < 1 cm diameter
*Grade 2*	Multiple, one or more > 1 cm, and all < 4 cm in diameter
*Grade 3*	Multiple, one or more > 4 cm

**Table 3:** Characteristics of the TSC patients and age, sex, BMI, and diabetes status matched controls. ALT: alanine transaminase; AST: aspartate transaminase; BMI: body mass index; DBP: diastolic blood pressure; eGFR: estimated glomerular filtration rate; HDL-C: high-density lipoprotein cholesterol; LDL-C: low-density lipoprotein cholesterol; mTOR: mammalian target of rapamycin; Non-HDL-C: non-high-density lipoprotein cholesterol; SBP: systolic blood pressure; TGL: triglycerides; Total C: total cholesterol. The diabetes status is defined as the presence of diabetes type 1 or 2; Alcohol abuse is defined as ≥30 g/day for men and ≥20 g/day for women; Present medication intake: the number of patients and controls that took medication at the time of the abdominal MRI; Past medication intake: the number of patients and controls that took medication before the abdominal MRI for more than 6 months. The significance level was 0.05. Significant *p*-values are presented in bold.

*Variable*	*TSC Patients* *N = 59*			*Controls* *N = 59*			
	N (%)	Mean (SD)	Median (IQR)	N (%)	Mean (SD)	Median (IQR)	*p*-Value
*Age* (years)		24.7 (17.6)	18.6 (27.9)		24.7 (17.0)	19.0 (28.2)	
*Male*	21 (35.6)			21 (35.6)			
*Female*	38 (64.4)			38 (64.4)			
*BMI* (kg/m^2^)		23.0 (5.7)	23.5 (9.3)		22.9 (5.7)	23.2 (9.9)	
*Abdominal circumference on MRI* (cm)		81.2 (19.6)	82.7 (32.5)		77.6 (17.6)	82.5 (30.3)	**0.019**
*SBP* (mmHg)		115.5 (24.4)	115.0 (26.5)		118.2 (18.3)	119.0 (24.0)	0.177
*DBP* (mmHg)		68.2 (18.5)	70 (14.5)		73.8 (13.3)	75.0 (15.3)	0.177
*eGFR* (mL/min/1.73 m^2^)		105.2 (23.9)	107.0 (37.8)		109.3 (20.5)	110.0 (34.5)	0.735
*AST* (units/L)		26.7 (20.8)	22.0 (12.0)		51.2 (69.7)	28.0 (20.5)	**0.003**
*ALT* (units/L)		28.4 (40.3)	20.0 (15.8)		76.5 (147.2)	21.0 (22.0)	**0.017**
*HDL-C* (mg/dL)		53.7 (12.0)	53.0 (15.0)		53.4 (30.4)	47.5 (21.5)	0.600
*LDL-C* (mg/dL)		107.9 (32.5)	107.0 (39)		167.5 (232.7)	104.0 (56)	0.875
*TGL* (mg/dL)		130.9 (156.8)	88.0 (88.0)		92.4 (38.9)	90.5 (64.0)	0.256
*Total C* (mg/dL)		178.3 (50.0)	172.0 (42.0)		159.0 (32.8)	159.0 (42.0)	0.078
*Non-HDL-C* (mg/dL)		130.3 (47.6)	124.0 (36.5)		115.2 (36.4)	113.0 (48.0)	0.241
*Diabetes status*	1 (1.7)			1 (1.7)			
*Alcohol abuse*	0.0 (0.0)			0.0 (0.0)			1.000
*Present medication intake:*							
*mTOR inhibitors*	16 (27.1)			0.0 (0.0)			**0.01**
*anti-epileptics*	30 (50.8)			1 (1.7)			**0.01**
*antihypertensive medication*	5 (8.5)			2 (3.4)			0.453
*Past medication intake:*							
*mTOR inhibitors*	4 (6.8)			0.0 (0.0)			0.125
*anti-epileptics*	19 (32.2)			1 (1.7)			**0.01**
*antihypertensive medication*	1 (1.7)			0.0 (0.0)			1.000

**Table 4:** Characteristics of the TSC patients. TSC1: tuberous sclerosis complex type 1; TSC2: tuberous sclerosis complex type 2. The major criteria are used for the clinical diagnosis of TCS. A definite diagnosis of TSC is made when two major criteria or one major criterion with two minor criteria are present (16). Diagnosis can also be made based on a pathogenic variant in TSC1 or TSC2. All patients had a definitive diagnosis of TSC.

*Major Criteria*	N (%)
*Hypomelanotic macules (≥3, at least 5 mm diameter)*	34 (57.6)
*Angiofibroma (≥3) of fibrous cephalic plaque*	39 (66.1)
*Ungual fibromas (≥2)*	8 (13.6)
*Shagreen patch*	19 (32.2)
*Multiple retinal hamartomas*	13 (22)
*Multiple cortical tubers and/or radial migration lines*	56 (94.9)
*Subependymal nodule (≥2)*	39 (66.1)
*Subependymal giant cell astrocytoma*	13 (22)
*Cardiac rhabdomyoma*	12 (20.3)
*Lymphangiomyomatosis*	8 (13.6)
*Angiomyolipomata*	28 (47.5)
***Genetic Data***	**N (%)**
*TSC1*	13 (22)
*TSC2*	32 (54.2)
*No mutation found*	7 (11.9)
*Not Tested*	7 (11.9)

**Table 5:** Liver phenotype of TSC patients compared to age, sex, BMI, and diabetes-matched controls. A McNemar test was used to compare the proportion of liver steatosis and a Wilcoxon signed ranks *t*-test was used to compare the percentage of liver steatosis in both groups. IQR: interquartile range; SD: standard deviation. In the top row, the number of patients with liver steatosis, defined as a fat signal fraction ≥5% on MRI, is compared to the controls. In the bottom row, the mean and median percentage of liver steatoses are compared between both groups. The significance level was 0.05.

	*TSC Patients* *N = 59*	*Controls* *N = 59*	*p-Value*
	N (%)	N (%)	
*Proportion of liver steatosis*	9 (15.3)	14 (23.7)	0.267
	Median (IQR)	Median (IQR)	
*% of liver steatosis*	2.0 (2.0)	2.0 (2.0)	1.000

**Table 6:** Conditional logistic regression on patients with TSC and matched controls. The first line represents the odds ratio of liver steatosis in patients with TSC. The second line reports the odds ratio of liver steatosis in patients with TSC after adjustment for the current use of mTOR inhibitors. The last line describes the odds ratio of liver steatosis in patients with TSC after adjustment for current use of medication (mTOR inhibitors, anti-hypertensive medication, anti-epileptic medication). TSC: tuberous sclerosis complex; mTORi: mechanistic target of rapamycin inhibitor; Med. intake: medication intake.

*Liver Steatosis*	Adjustment for	Odds Ratio (95% CI)	*p*-Value
*TSC*	Univariate	0.44 (0.14 to 1.44)	0.18
	mTORi	0.75 (0.17 to 3.35)	0.71
	Medication intake	0.46 (0.14 to 1.50)	0.20

**Table 7:** Characteristics of liver angiomyolipomata in patients with TSC. SD: standard deviation; IQR: interquartile range. Grade 0: No liver angiomyolipomata; Grade 1: One or more liver angiomyolipomata, all < 1 cm diameter; Grade 2: Multiple liver angiomyolipomata, one or more > 1 cm diameter, and all < 4 cm in diameter; Grade 3: Multiple liver angiomyolipomata, one or more > 4 cm. Two patients with liver angiomyolipomata could not be graded since they only had one angiomyolipoma that was >1 cm. The mean and median diameter of the biggest angiomyolipoma is represented in the bottom row.

	*TSC Patients* *N = 59*		
	N (%)		
*Grade 0*	51 (86.4)		
*Grade 1*	5 (8.5)		
*Grade 2*	1 (1.7)		
*Grade 3*	0 (0)		
*No gradation*	2 (3.4)		
		Mean (SD)	Median (IQR)
*Maximum diameter* (cm)		0.9 (0.5)	0.7 (0.6)

## Data Availability

Available data not included in the current manuscript will be made available by the authors upon substantiated request.

## Supplementary Materials

The following supporting information can be downloaded at: https://www.mdpi.com/article/10.3390/jcm13102888/s1, Table S1: Clinical diagnostic criteria of TSC; Table S2: Factors studied for association with liver steatosis; Table S3: Factors studied for association with liver angiomyolipomata in patients with TSC; Table S4: Overview of the indications and conclusions of the abdominal MRI in the control population; Text: Selection procedure for TSC patients and control group matching.
