# Supplementary material for: Prevalence of Liver Steatosis in Tuberous Sclerosis Complex Patients: A Retrospective Cross-Sectional Study
Source: J Clin Med. 2024 May 14;13(10):2888. doi: 10.3390/jcm13102888 (PMC11122077; doi:10.3390/jcm13102888)
Supplement: Supplementary file 1 [file jcm-13-02888-s001.zip › jcm-2984153-supplementary.pdf]

Supplementary data

Clinical criteria for diagnosis of TSC

**Major criteria**	**Minor criteria**
Hypomelanotic macules (≥3, at least 5 mm diameter)	'confetti' skin lesions
Angiofibroma (≥3) of fibrous cephalic plaque	Dental enamel pits (≥3)
Ungual fibromas (≥2)	Intraoral fibromas (≥2)
Shagreen patch	Retinal achromic patch
Multiple retinal hamartomas	Multiple renal cysts
Multiple cortical tubers and/or radial migration lines	Nonrenal hamartomas
Subependymal nodule (≥2)	Sclerotic bone lesions
Subependymal giant cell astrocytoma	
Cardiac rhabdomyoma	
Lymphangiomyomatosis	
Angiomyolipomas	

Table S1: Clinical diagnostic criteria of TSC. A definite diagnosis of TSC can be made when 2 major features or 1 major feature with 2 minor features are present. TSC is possible when either 1 major feature or more than 2 minor features are present (1)

Selection procedure for TSC patients and control group matching

The control group was matched with TSC patients according to their age, sex, BMI, and diabetes status. To limit bias, the selection procedure was pre-defined:

1) Selection of control subjects from the database based on sex and age, using age intervals of 3 years.
2) Ranking sex-matched subjects based on the date of the imaging.
3) Selection of subjects based on age (closest matched) at imaging.
4) Assessment of BMI (+/- 1 kg/m^2 within 1.5 years of the date of imaging).
5) Assessment of diabetes status (according to the type of diabetes and whether the diabetes is controlled or uncontrolled).
6) Assessment of additional exclusion criteria (see Table 1). If no additional exclusion criteria are met, the subject was included as a control.

If excluded, the following potential control subject was the closest age match, and the procedure was repeated. Only when the selection of patients and controls was finished, liver imaging and other parameters were assessed.

Potential associated factors with liver steatosis and liver angiomyolipomata

**Factors explored for association with liver steatosis**	
Age	GGT
Gender	AST
Abdominal circumference	ALT
BMI	Platelets
Type of TSC mutation	Triglycerides
Fasting glucose	HbA1c
CRP	eGFR
HDL-cholesterol	Systolic blood pressure
LDL-cholesterol	Diastolic blood pressure
Hypertension	Diabetes status
Current medication: systemic mTOR inhibitors, anti-epileptics, antihypertensive medication	Past medication taken >6 months: systemic mTOR inhibitors, anti-epileptics, antihypertensive medication
Alcohol abuse	

Table S2: Factors studied for association with liver steatosis. ALT: alanine transaminase; AST: aspartate transaminase; BMI: body mass index; CRP: C-reactive protein; eGFR: estimated glomerular filtration rate; GGT: gamma-glutamyl transferase; HbA1c: hemoglobin A1c; HDL: high-density lipoprotein; LDL: low-density lipoprotein; mTOR: mammalian target of rapamycin; TSC: tuberous sclerosis complex. Alcohol abuse is defined as ≥30 g/day for men and ≥20 g/day for women.

Factors explored for association with liver angiomyolipomata	
Age	Type of TSC mutation
Gender	Presence of angiomyolipomas in the kidneys
Abdominal circumference	AST
BMI	ALT
Current medication: systemic mTOR inhibitors, anti-epileptics, antihypertensive medication<br>Past medication taken >6 months: systemic	Past medication taken >6 months: systemic mTOR inhibitors, anti-epileptics, antihypertensive medication
Alcohol abuse	

Table S3: Factors studied for association with liver angiomyolipomas in patients with TSC. ALT: alanine transaminase; AST: aspartate transaminase; BMI: body mass index; mTOR: mammalian target of rapamycin; TSC: tuberous sclerosis complex. Alcohol abuse is defined as ≥30 g/day for men and ≥20 g/day for women.

Overview of the indications and conclusions of the abdominal MRI in the control group

Controle record ID	AST (U/l)	ALT (U/l)	% of liver steatosis	Reason of MRI	Conclusion MRI
17	30	25	2	Abdominal mass is seen on echo.	Neuroblastoma.
18	32	18	2	Histiocytosis from skin biopsy.	No intra-abdominal adenopathies.
19	45	15	2	Dilated bile ducts on ultrasound.	Lithiasis at the level of the pancreatic head.
20	28	21	2	Control of an abdominal mass.	Echinococcus cyst versus amoebic abscess.
21	35	18	2	Suspected Wilms tumor, metastasis?	Wilms tumor.
22	36	36	2	Suspected nephroblastoma on ultrasound abdomen.	Lesion suspected for nephroblastoma.
23	27	37	8	Intermittent joint pain and ulcers, DD Behcet, or vasculitis?	No arguments for Behcet or vasculitis.
24	46	28	2	Abdominal mass is seen on echo.	Abscess as a complication of perforated appendicitis.
25	30	37	10	pancreatic atrophy? Evaluation of renal cysts.	Pancreatic body and tail atrophy. Multiple cysts in kidney parenchyma.
26	30	37	5	Lipoma abdominal wall?	Non-encapsulated lipoma.
27	30	35	11	Follow-up pancreatitis, zone of necrosis at the level of corpus tail section.	Multiple hemorrhagic components are anterior to the pancreas. A small pseudocyst is seen cranial to the pancreatic tail.
28	**300**	**116**	2	Septic emboli? High fever, high CRP, and abdominal pain.	Image compatible with scattered millimetric micro-abscesses in the liver.
29	/	/	2	Hirsutism.	Image compatible with polycystic ovaries.
30	/	/	2	Abdominal pain. Adrenal glands?	Normal adrenal glands.

31	/	/	1	Mass at the level of the left kidney upper pole on duplex kidneys. Significant pyuria and infection without symptoms.	Known duplicated pyelocalicial system on the left with moderate hydronephrosis. No evidence of suspicious masses.
32	20	18	3	Inflammatoire appendix?	The image may be compatible with a pus-filled appendix.
37	27	15	2	Follow-up chronic pancreatitis.	No arguments for any pancreatitis or consequences of previous pancreatitis.
38	17	8	2	Slightly increased lipase. Pancreatic lesion? IPMN?	Examination within normal limits.
39	30	37	2	Young age pancreatitis, pancreatic divisum, cholecystolithiasis?	Normal image.
40	15	12	2	Hyperandrogenism.	No notable abnormalities.
51	20	14	2	Status post cholecystectomy, choledocholithiasis? Cholestasis.	No choledocholithiasis.
52	11	15	25	Hypodens nodule on the pancreas.	Scattered biliary cysts in the liver. No pancreatic pathology.
53	**53**	**59**	2	Recurrent acute pancreatitis.	Normal pancreas. No anatomic abnormalities.
54	34	39	2	Vomiting and diarrhea, primary biliary cholangitis, cholestasis, cholelithiasis? Status primary biliary cholangitis?	Normal imaging post-cholecystectomy.
55	12	10	3	Follow-up NF1.	No suspicious lesions. Smoothly delineated nodule in the muscle of abdominal wall right lateral, not diffuse. Preferred diagnosis: neurofibroma.
56	22	17	1	Accidental finding of cyst on ultrasound.	Cyst at the level of the pancreatic body, with probable connection to

					the Wirsung: then it is a side branch IPMN.
57	18	20	6	Increased androgens.	No adrenal nodules.
58	25	33	3	Ulcerative colitis and PSC with treatment-dominant stricture.	Multiple stenosis at the level of the intrahepatic bile ducts with slight dilatation. There is a widened appearance of the ductus choledochus. Staining of the wall: post manipulation?
59	19	20	2	MEN1 for which in the past operation.	Stable aspect of cysts at the level of residual pancreatic parenchyma. Multiple millimetric cholecystolithiases.
60	22	31	1	Insulinoma?	No suspected insulinoma lesion.
71	12	10	5	Rule out lithiasis or sludge.	Sludge in gall bladder. No other abnormalities.
72	15	19	7	Hydrops of gallbladder, dilated bile ducts, mildly impaired liver function tests; ERCP normal, anatomical abnormalities bile ducts? Lithiasis? Neoplasia?	Sludge in gall bladder. Normal bile ducts. No focal suspected lesions.
73	23	21	3	Cowden syndrome screening: risk of pancreas carcinoma.	No evidence of suspicious intra-abdominal lesions.
74	20	24	9	Minor lesions at the level of the pancreatic head.	No suspected lesions in the pancreas. Normal aspect of the other abdominal organs.
75	19	15	25	Injury to upper pole left kidney. Evolution?	Fatty nodule at the upper pole of the left kidney, consistent with a small angiomyolipoma.
76	32	20	2	Evaluation operability cholangiocarcinoma.	Extensive tumoral lesions in the liver. Confirmation of the presence of a

					suspicious lesion in proximal colon descendens.
77	**236**	**578**	1	Dilated ductus choledochus.	No dilated bile ducts. No choledocholithiasis.<br>Multiple gallbladder lithiasis.
78	33	**60**	6	Follow-up NF1.	Splenomegaly. Numerous millimetric nodules mostly in the skin, neurofibromatosis.
79	**108**	**405**	2	Evaluation of bile ducts, bile duct lithiasis? Wall thickening? PSC? Limited inflammation, hyperbilirubinemia.	No gallbladder lithiasis nor bile duct lithiasis. Image of (relapsed) cholecystitis with underlying sludge versus hemorrhage.<br>Dilated intrahepatic bile ducts without stop image. Regularly widened bile ducts, making a PSC unlikely.
80	**107**	**534**	3	Post-operative laparoscopic cholecystectomy 11/2021. Recent recurrence of symptomatic bile colic with accompanying cholestatic laboratory. Persistent bilirubinemia and especially ascending AF and ALT. Common bile duct free?	Choledocholithiasis of 8 mm.
100	42	**258**	1	Pancreatitis with biochemical stigmata of lithiasis. Complicated pancreatitis? Choledocholithiasis?	Acute pancreatitis with infiltration of adjacent fat. Cholecystolithiasis without signs of cholecystitis. No bile duct obstruction.
101	28	13	3	On previous ultrasound hyperintensities spleen. Aspect on MRI? Calcifications or other? Psychomotor retardation and	Confirmation of a solitary lesion in the spleen, with no suspicious features. The preferred diagnosis is splenic hamartoma.

				hyperinsulinism in complex translocation chromosome 9 and 11.	
102	**190**	54	5	Abscess? Appendicitis? Enteritis? Urinary tract? Anomaly in the small pelvis? Lower abdominal pain and diarrhea, fever, under antibiotics, severe inflammatory blood count.	Hepatomegaly, ascites fluid, interstitial edema in the lungs. Image primarily fits a multisystem inflammatory disorder. Clinically correlatable. Also, notice fat infiltration around the right ureter. Ascending urinary tract infection is not excluded.
103	**66**	**98**	3	Choledocholithiasis? History of biliary pancreatitis.	Cholecystolithiasis. No choledocholithiasis. Acute exudative pancreatitis.
104	/	/	0	Weight loss, epigastric pain, no longer able to tolerate fat.	No signs of malignancy.
105	/	30	2	Oestrogenisation of unknown origin. Adenoma?	Normal adrenal glands.
106	40	/	1	Follow-up primary sclerosing cholangitis. Ulcerative colitis.	Known PSC without evidence of acute flare-up. Gallbladder lithiasis of 8 mm.
107	30	**57**	2	Urinary tract lesion or arteriovenous malformation? Macroscopic haematuria.	Normal morphology of both kidneys except for a small cortical renal cyst at the level of the left kidney lower pole.
108	40	18	2	Newly diagnosed neuroblastoma.	Voluminous grape-shaped mass from the posterior mediastinum to the aortic bifurcation. No evidence of vascular ingrowth. Normal appearance of liver, spleen, and

					kidneys. Image suspicious for diffuse bone metastasis.
109	**200**	**390**	5	Cholelithiasis. Also stones in the bile ducts? Cholangitis? External obstruction?	Gallbladder lithiasis. No signs of cholecystitis. Image compatible with bile duct lithiasis in the most distal part of the choledocus with dilatation of intrahepatic bile ducts in the left and right hepatic lobe.
110	17	21	1	Chronic abdominal pain. Check the kidneys, abdomen, and ovaries. PCOS?	Normal imaging.
111	**328**	**655**	3	Bile colic. Choledocholithiasis?	Dilatation of the intrahepatic bile ducts and ductus choledochus due to two distal choledocholithiases. Multiple substantial cholecystolithiases. No evidence of cholecystitis.
112	19	14	3	On ultrasound cyst in the spleen. Type? Abnormalities? Complaints of centro-abdominal abdominal pain.	Image of simple epithelial cyst in the spleen. Differentially diagnostic: this may also be a hydatid cyst.
113	18	14	2	Hypoglycemia.	No focal pancreatic lesions.<br>Focal nodular hyperplasia of 4 cm, in liver segment 1.
114	13	10	4	Follow-up hemangioma.	Image best fitting for haemangioma in liver segment VI.
115	/	18	13	Choledocolithiasis?	Lithiasis of the gall bladder.
116	22	22	3	PSC to be ruled out, other biliary pathology? Mild LFT abnormalities with an IBD-like story.	Normal findings. In particular, there are no arguments for a PSC or other biliary pathology.
117	21	17	1	Dilated bile ducts. Symptomatic bile colic.	Sludge in the gallbladder, otherwise no biliary pathology.

118	22	14	2	Hydronephrosis on ultrasound. High CRP. Appendicitis?	No evidence of appendicitis. Pronounced pyelonephritis of the right kidney with dilated pyelum and ureter to the overcrossing of the great vessels.

Table S4: Overview of the indications and conclusions of the abdominal MRI in the control population. The AST and ALT values as well as the percentage of liver steatosis were included in the table. AF: alkaline phosphatase; ALT: alanine transaminase; AST: aspartate transaminase; CRP: C-reactive protein; DD: differential diagnosis; ERCP: Endoscopic retrograde cholangiopancreatography; IBD: irritable bowel disease; LFT: liver function tests; MEN1: Multiple endocrine neoplasia type 1; NF1: Neurofibromatosis type 1; PCOS: polycystic ovary syndrome; PSC: Primary sclerosing cholangitis. The upper limit of normal for ALT is 55 U/L and AST 48 U/L. Abnormal AST and ALT values are highlighted in bold.

References

1. Northrup H, Aronow ME, Bebin EM, Bissler J, Darling TN, de Vries PJ, et al. Updated International Tuberous Sclerosis Complex Diagnostic Criteria and Surveillance and Management Recommendations. Pediatr Neurol [Internet]. 2021 Oct 1 [cited 2022 Apr 11];123:50–66. Available from: https://pubmed.ncbi.nlm.nih.gov/34399110/
